# Immunisation timeliness in a cohort of urban Aboriginal and Torres Strait Islander children

**DOI:** 10.1186/s12889-016-3825-z

**Published:** 2016-11-14

**Authors:** Yolanda G. Lovie-Toon, Kerry K. Hall, Anne B. Chang, Jennie Anderson, Kerry-Ann F. O’Grady

**Affiliations:** 1 Institute of Health & Biomedical Innovation, Queensland University of Technology, Centre for Children’s Health Research, South Brisbane, QLD Australia; 2 Menzies School of Health Research, Charles Darwin University, Tiwi, NT Australia; 3 Respiratory Department, Lady Cilento Children’s Hospital, South Brisbane, QLD Australia; 4 Murri Health Group, Caboolture, QLD Australia

**Keywords:** Indigenous, Aboriginal, Australia, Childhood, Paediatrics, Immunisation, Timeliness

## Abstract

**Background:**

To evaluate immunisation coverage, timeliness and predictors of delayed receipt in urban Australian Indigenous children during the first 18 months of life.

**Methods:**

Cross-sectional retrospective analysis of data collected from 140 Australian Indigenous children aged < 5 years at the time of enrolment in a prospective cohort study on respiratory illness between 14 February 2013 and 28 January 2015. Children were recruited through an urban community primary health care centre in the Northern suburbs of Brisbane, Queensland.

**Results:**

The proportion of children with completed immunisation schedules was 50 of 105 (47.6%) at 7 months, 30 of 85 (35.3%) at 13 months and 12 of 65 (18.5%) at 19 months. Timely receipt of diphtheria-tetanus-pertussis decreased from 78.4% at 2 months of age to 63.7 and 59.3% at 4 and 6 months respectively. Amongst the 105 parents/guardians with children ≥7 months at enrolment, 71 (67.6%) incorrectly reported their child’s immunisation status. Delayed vaccine receipt was significantly associated (p ≤0.05) with having multiple children in the household, mother’s unemployment and premature birth.

**Conclusions:**

Coverage and timeliness among this population is suboptimal and decreases as children age. Parent/guardian reporting of vaccination status was unreliable. Children of unemployed mothers and those with multiple siblings should be targeted to improve community immunisation timeliness due to a greater risk of vaccination delay. High quality trials, conducted in several settings to account for the diversity of Australian Indigenous communities are urgently needed to identify culturally appropriate, effective and sustainable strategies to improve immunisation targets in children.

## Background

Vaccine timeliness, important for early individual protection and generation of herd immunity [1], is particularly crucial in infancy [2] and settings with high infection rates (e.g. Indigenous communities). Australian Indigenous Australian children experience higher notification and hospitalisation rates for vaccine preventable infections and diseases than non-Indigenous children [3–6]. Furthermore, some infections pose a greater risk of severe disease and adverse outcomes when contracted during infancy and early childhood [3].

In 2013, national vaccination coverage at 12, 24 and 60-months was 86.0, 92.0 and 93.0% respectively among Indigenous children, and 90.6, 92.1 and 91.6% among non-Indigenous children [5]. Although these are applauded, reporting coverage at these milestones can conceal overdue vaccination occurring before these milestones [7, 8] and data reported at a national level does not account for the heterogeneity of Australian Indigenous communities. A number of Australian studies have now highlighted the gap in timely immunisation between Indigenous and non-Indigenous children, including regional differences, over the past decade [4, 7, 9, 10]. The most current data indicates that the gap in timeliness remains, with the proportion of delayed receipt being 5.8-21.6 percentage points higher among Indigenous children compared to non-Indigenous children for selected vaccines (3rd dose of diphtheria-tetanus-pertussis (DTP) and 1st and 2nd dose of measles-mumps-rubella (MMR) [5]. However there are limited current data on whether this gap exists among other vaccines and at earlier time points. There are also little data on differences in coverage within and between Indigenous communities and on predictors of incomplete coverage and timeliness in this population that account for its diversity. Availability of such data would inform a targeted approach and potentially influence policy in our era of limited health resources.

Our primary objective was to evaluate vaccination coverage and timeliness during the first 18-months of life among a cohort of urban Indigenous children. Our secondary objectives were to: a) identify potential predictors of delayed vaccination, and; b) evaluate the validity of parent/carer report of child immunisation status.

## Methods

This study was conducted in an urban community primary health care centre in the Northern suburbs of Brisbane, Queensland. The centre opened in 2011 and has over 10,000 registered clients. Approximately 65% identify as Aboriginal and/or Torres Strait Islander, which approximates to 14% of the Brisbane’s Indigenous population based on the most recent census data [11].

This paper reports on results from a retrospective analysis of children at the time of enrolment into a prospective cohort study of paediatric acute respiratory illnesses (ARI) currently underway at the centre [12]. Children presenting to the centre for any reason, including accompanying another person, are invited to participate. Children are eligible for participation if they are aged < 5 years at time of enrolment, registered as a patient with the clinic and intend to remain in the study area for the following 12 months. Written consent is obtained from the parent(s)/guardian(s) before study entry. Ethical approval was obtained from the Queensland Children’s Health Services (HREC/12/QRCH/169) and the Queensland University of Technology (1300000741) Human Research Ethics Committees. Additionally, this study was conducted in accordance with the guidelines for ethical conduct in Aboriginal and Torres Strait Islander Health Research outlined by the Australian National Health and Medical Research Council [13] and the cultural oversight was provided by an Indigenous Reference Group. Indigenous children enrolled prior to 28 January 2015 were included in this analysis. As per national standards, Indigenous status was determined through self-report [14]. Of all the children aged < 5 years registered in the clinic during the study period, 16.3% participated in this study.

Parent/guardian-reported demographic, infant, maternal and paternal characteristics were collected at baseline. Parent/guardian report of their child’s immunisation status was collected by asking “Is your child age appropriately immunised for childhood vaccines?”. With parent/guardian consent, immunisation records were retrieved from the Australian Childhood Immunisation Register (ACIR). ACIR is a nationwide opt-out register characterised by incentivised reporting of vaccine administration by immunisation providers. Inclusion in ACIR is based on one’s enrolment in Australia’s universal health care scheme, of which 99% of Australians are enrolled in by 12 months of age [5].

During the period in which the study participants were born (2008–2014), there were multiple changes to the Immunisation Program Schedule for Queensland [15]. Therefore our analysis was conducted according to antigens, opposed to vaccines. Only those antigens that were recommended for Indigenous children at the same time points across all five schedules (Table 1) were included in our analysis. The series of vaccines due at 2, 4 and 6 months are henceforth referred to as the ‘primary series’ of vaccines.

**Table 1:** Antigens recommended for Indigenous children across all QLD Immunisation Program Schedules used from 2008–2015

Age vaccine is due	Antigen
2, 4 & 6 months	Diphtheria, Tetanus, Pertussis (DTP)
	Polio
	Hepatitis B (HepB)
	*Haemophilus influenza* type b (Hib)
	Rotavirus (Rota)
	Pneumococcal (PCV)
12 months	Measles, mumps, rubella (MMR)
	*Haemophilus influenza* type b (Hib)
	Meningococcal C (MenC)
18 months	Varicella
	Hepatitis A (HepA)

In Queensland, the majority of vaccines administered to infants and young children are administered by general practitioners through primary health care centres. Some local government areas also provide public immunisation clinics. Vaccines given at birth (e.g. Bacillus Calmette–Guérin) are routinely received through hospitals and frequently recorded in a different system to ACIR. Therefore birth vaccines were excluded from all analyses.

Immunisation coverage and timeliness was calculated using only those vaccines received prior to or on the date of enrolment. Children were included in the coverage and timeliness calculations at each milestone if, at their date of enrolment, they were ≥1 month older than the respective milestone. This allowed for vaccines administered within 1 month of the vaccine due date to be included in the analyses.

The Australian National Centre for Immunisation Research and Surveillance routinely allows a 3-month lag period between vaccine receipt and report to ACIR [5]. Therefore, we only analysed vaccines that were due to have been received ≥3 months prior to the date we extracted immunisation records from ACIR. Immunisation timeliness was classified according to the differences in weeks between the dates at which vaccines were due and actually received. There is no universally accepted definition of vaccine timeliness. Australian studies typically define timely receipt as received ≤30 days after the vaccine due date and delayed as >30 days after the due date [5, 7, 10]. Therefore timeliness was classified using the following definitions:

- Early (received >14 days before due date)
- No delay (received between 14 days before and 30 days after due date)
- Delayed (received >30 days after the due date)
- Not received (no recorded dose prior to enrolment date)
- Incorrectly received (vaccine received when not due/eligible)

The third dose assumption was not applied to the treatment of ACIR data in this study. This assumes that where there is a record of a second or third dose of a vaccine being received the previous dose has also been received, regardless of whether there is a record of it [16]. We assumed that the first record of any vaccination on ACIR was the first dose and checked the effect of that assumption by evaluating the number of children for whom a third dose assumption would have been applied if we had taken that approach.

### Statistical analysis

Child characteristics at enrolment and vaccination data were tabulated and expressed as proportions and/or mean/medians with the corresponding 95% Confidence Intervals (CI) or Interquartile Ranges (IQR). To identify potential predictors of vaccination delay, exploratory univariate analyses were performed with timeliness recoded as a dichotomous variable with ‘delayed’ or ‘not received’ being classified as ‘delayed’, and ‘early’ or ‘no delay’ being classified as ‘no delay’. Children with responses of ‘unknown’, ‘not applicable’ or ‘declined to answer’ for any of the predictor variables were excluded from the analysis. The predictor variables ‘participating health care service is usual provider’ and ‘multiple birth’ were excluded from the analyses as more than 90% of participants reported the same response. The analysis of predictors of timeliness was only performed for the DTP vaccines, given its consistency in the primary series across all vaccine schedules. A two-tailed *p*-value of <0.05 was considered significant. All analyses were performed in Stata V13 (StataCorp, Texas, USA).

## Results

Between 14 February 2013 and 28 January 2015, 140 children were enrolled; median age at enrolment of 18.3 months (IQR 6.9–32.0). ACIR records were unavailable for four participants who were excluded from further analyses. Of the remaining 136 children, 71 (50.7%) were male. Child characteristics and that compared to national Aboriginal and Torres Strait Islander population (where data are available) are provided in Table 2.

**Table 2:** Demographic, infant, maternal and paternal characteristics of study population

Characteristic	N = 140 n (%)	National Indigenous population (%)
Sex		
Male	71 (50.7)	50.9 [27]
Female	69 (49.3)	49.1 [27]
Care type		
Both parents	80 (57.1)	54.7 [28]
Single parent	51 (36.4)	45.3 [28]
Other^a^	8 (5.7)	
Missing	1 (0.7)	
Primary carer		
Mother	115 (82.1)	
Other^b^	16 (11.4)	
Missing	9 (6.4)	
Childcare^c^		
Yes	35 (25.0)	21.7^f^ [29]
No	104 (74.3)	
Missing	1 (0.7)	
Study clinic is usual health care provider		
Yes	131 (93.6)	N/A
No	0 (0.0)	N/A
Missing	9 (6.4)	N/A
Educational level of father		
Did not finish high school	63 (45.0)	58.0^g^ [30]
High school	37 (26.4)	16.0^g^ [30]
Post-school qualification^d^	16 (11.4)	25.9^g^ [30]
Missing	24 (17.1)	
Educational level of mother		
Did not finish high school	54 (38.6)	
High school	66 (47.1)	
Post-school qualification^d^	16 (11.4)	
Missing	4 (0.3)	
Employment status of father		
Employed^e^	60 (42.9)	49.7^h^ [30]
Unemployed	57 (40.7)	50.3^h^ [17]
Missing	23 (16.4)	
Employment status of mother		
Employed^e^	17 (12.1)	43.0^h^ [30]
Unemployed	121 (86.4)	57.0^h^ [30]
Missing	2 (1.4)	
Income level		
< $26000	44 (31.4)	35.2^i^ [31]
$26000 - < $52000	56 (40.0)	34.3 ^i^ [31]
$52000 - < $78000	23 (16.4)	8.9^i^ [31]
≥ $78000	11 (7.9)	4.0^i^ [31]
Missing	6 (4.3)	
Multiple birth		
Singleton	134 (95.7)	98.5^j^ [32]
Multiple	1 (0.7)	1.5^j^ [32]
Missing	5 (3.6)	
	Mdn (IQR)	Mean/Mdn
Number of children in household (N = 138)	2.0 (3.0-1.0)	
Age of mother at birth of child (in years) (N = 138)	24.0 (29.0-20.0)	25.2^k^ [33]
Age of father at birth of child (in years) (N = 135)	26.0 (33.0-23.0)	27.9^k^ [33]
Gestational age (in weeks) (N = 137)	40.0 (40.0-38.0)	38.2^m^ [33]
Birth weight (in grams) (N = 137)	3200.0 (3628.0-2770.0)	3200 ^m^ [33]

^a^Other = shared, other relative, other non-family member (including government care); ^b^Other = Father, Mother and Father, Grandparent, Grandparent and Father, Other non-family; ^c^Childcare = formal regulated daytime care provided in a group setting, includes private or community centres and family day care; ^d^Post-school qualification = Certificate, Diploma, Bachelor Degree; ^e^Employed = Full Time, Part Time, Casual; ^f^Includes children aged 0–4 years exclusively attending formal childcare and children aged 0–4 years attending both formal and informal childcare; ^g^Data represents both males and females; ^h^Unemployed = those not participating in the labour force and those participating in the labour force but without work; ^i^Categories of income levels presented for national data differ from categories used in study and are as follows: <$20800, $20800- < $52000, $52000- < $78000, ≥$78000; ^j^Data represents all Australian women, not solely Indigenous Australian women; ^k^Median value; ^m^Mean value

Immunisation coverage at the selected milestones was low among all age groups, and decreased in older children (Table 3). To consider the effect of the upper age restriction of rotavirus vaccines on overall coverage, coverage was recalculated excluding this antigen. The increase in coverage through the exclusion of rotavirus was by less than 10 percentage points at each milestone.

**Table 3:** Coverage of children fully immunised at date of their enrolment

Age	Eligible children N	Children fully immunised n (%, 95% CI)	Children fully immunised, excluding rotavirus vaccine. n (%, 95% CI)
7 months^a^	105	50 (47.6, 37.9–57.3)	57 (54.3, 44.6–64.0)
13 months^b^	85	30 (35.3, 24.9–45.7)	35 (41.2, 30.5–51.6)
19 months^c^	65	12 (18.5, 8.8–28.2)	14 (21.5, 11.3–31.8)

^a^Fully immunised at 7 months = 3 doses of each of DTP, hib, polio, PCV, hepB and rotavirus; ^b^Fully immunised at 13 months = 3 doses of each of DTP, polio, PCV, hepB and rotavirus, 4 doses of hib, 1 dose of MMR and 1 dose of menC; ^c^Fully immunised at 19 months = 3 doses of each of DTP, polio, PCV, hepB and rotavirus, 4 doses of hib, 1 dose of MMR, 1 dose of menC, 1 dose of varicella and 1 dose of hepA

Vaccine receipt and timeliness are presented in Table 4 for each antigen and dose. The highest proportions of non- receipt were for the rotavirus vaccine due at 4 (26.7%) and 6-months (42.9%) and the HepA vaccine due at 18-months (23.1%). Among the primary series of immunisations, the proportion of the cohort who had received each vaccine decreased at each milestone.

**Table 4:** Proportions of vaccine receipt and timeliness amongst eligible children

Vaccine	Eligible children^a^ N (%)	Total not received N (%)	Total received N (%)	No delay N (%)	Early N (%)	Delayed N (%)	Median number of weeks delayed Mdn (IQR)
PCV 1 (2 months)	126 (90.0)	1 (0.8)	125 (99.2)	99 (79.2)	1 (0.8)	25 (20.0)	9.1 (17.6-1.3)
PCV 2 (4 months)	120 (85.7)	7 (5.8)	113 (94.2)	72 (63.7)	0 (0.0)	41 (36.3)	7.4 (16.4-3.4)
PCV 3 (6 months)	105 (75.0)	13 (12.4)	92 (87.6)	54 (58.7)	1 (1.1)	37 (40.2)	10.4 (26.1-5.1)
DTP, polio & HepB 1 (2 months)	126 (90.0)	1 (0.8)	125 (99.2)	98 (78.4)	1 (0.8)	26 (20.8)	9.0 (18.0-1.3)
DTP, polio & HepB 2 (4 months)	120 (85.7)	7 (5.8)	113 (94.2)	72 (63.7)	0 (0.0)	41 (36.3)	7.4 (16.4-3.4)
DTP, polio & HepB 3 (6 months)	105 (75.0)	14 (13.3)	91 (86.7)	54 (59.3)	1 (1.1)	36 (39.6)	10.5 (24.9-3.9)
Hib 1 (2 months)	126 (90.0)	1 (0.8)	125 (99.2)	98 (78.4)	1 (0.8)	26 (20.8)	9.0 (18.0-1.3)
Hib 2 (4 months)	120 (85.7)	7 (5.8)	113 (94.2)	72 (63.7)	0 (0.0)	41 (36.3)	7.4 (16.4-3.4)
Hib 3 (6 months)	105 (75.0)	14 (13.3)	91 (86.7)	54 (59.3)	1 (1.1)	36 (39.6)	10.5 (24.9-3.9)
Rota 1 (2 months)	126 (90.0)	19 (15.1)	107 (84.9)	96 (89.7)	1 (0.9)	10 (9.3)	1.4 (12.7-1.0)
Rota 2 (4 months)	120 (85.7)	32 (26.7)	88 (73.3)	70 (79.5)	0 (0.0)	18 (20.5)	2.9 (5.9-1.0)
Rota 3 (6 months)	105 (75.0)	45 (42.9)	60 (57.1)	49 (81.7)	1 (1.7)	10 (16.7)	1.4 (2.3-0.7)
MMR (12 months)	85 (60.7)	3 (3.5)	82 (96.5)	38 (46.3)	0 (0.0)	44 (53.7)	7.4 (15.9-3.4)
MenC (12 months)	85 (60.7)	4 (4.7)	81 (95.3)	38 (44.7)	0 (0.0)	43 (50.6)	7.3 (15.0-3.4)
Hib 4 (12 months)	85 (60.7)	13 (15.3)	72 (84.7)	36 (50.0)	0 (0.0)	36 (50.0)	5.8 (10.7-3.1)
Varicella (18 months)	65 (46.4)	6 (9.2)	59 (90.8)	28 (47.5)	3 (5.1)	28 (47.5)	12.8 (42.3-2.9)
HepA (18 months)	65 (46.4)	15 (23.1)	50 (76.9)	13 (26.0)	1 (2.0)	36 (72.0)	19.2 (37.7-4.1)

^a^Eligibility for 2 month vaccines = child ≥ 3 months of age at enrolment, eligibility for 4 month vaccines = child ≥ 5 months of age at enrolment, eligibility for 6 month vaccines = child ≥ 7 months of age at enrolment, eligibility for 12 month vaccines = child ≥ 13 months of age at enrolment, eligibility for 18 month vaccines = child ≥ 19 months of age at enrolment

**Fig. 1:**
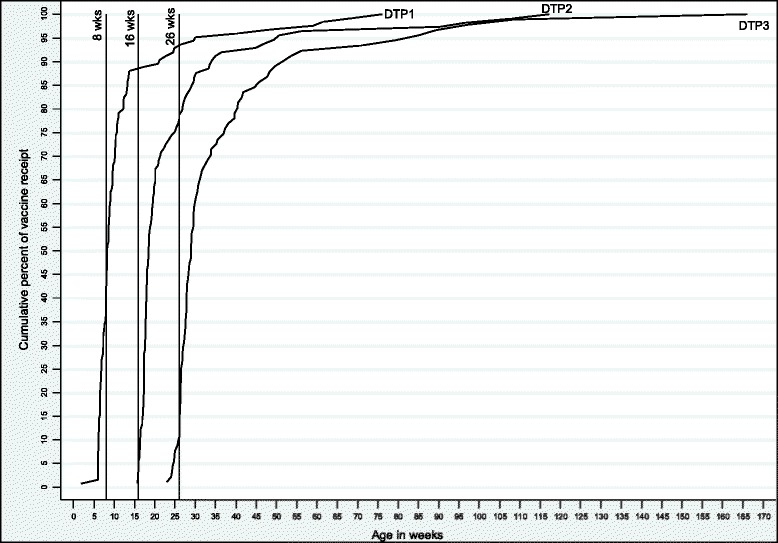
Cumulative percent of receipt of DTP due at 2, 4, 6 months among eligible children

The proportion of children who received a vaccine earlier than recommended was ≤2% for most vaccines and 5.1% for the varicella vaccine at 18-months (Table 4). Among those children who received the primary series, most (58.7–89.7%) received them with no delay (Fig. 1); this was not the case at 12 and 18-months of age. MMR due at 12-months and HepA due at 18-months had the highest rates of delayed receipt (53.7 and 72.0% respectively). Interestingly, there were notable variations in the proportions of receipt and timely receipt among the three vaccines due at 12 months, and more pronounceably among the two vaccines due at 18 months. Rotavirus vaccinations at 2, 4 and 6-months had the lowest rates of delayed receipt (9.3, 20.5 and 16.7% respectively). For those vaccines that were delayed, the median number of weeks delayed ranged from 1.4 to 19.2 weeks, with rotavirus vaccines having the shortest median delay and vaccines due at 18 months having the longest median delay. There were 6 instances where vaccinations were ‘incorrectly received’ either because the child was not due or was no longer eligible to receive the vaccine. The majority of these instances (4/6) were due to rotavirus being administered outside the recommended age cut-offs.

The majority of parents/guardians with children aged ≥7-months at enrolment reported that their child was up-to-date (96/105, 91.4%). However parent/guardian report was not consistent with ACIR; 71.9% of parents/guardians with who considered their child was up-to-date were incorrect compared to ACIR. Sensitivity and specificity of parental report was 93.1 and 9.2% respectively.

Univariate unadjusted analyses of the associations between child characteristics and delayed receipt of DTP at 2, 4 and 6-months, as well as delayed receipt at any time point, are presented in Table 5. There was a significant association (*p* < 0.05) between delayed receipt and the mother being unemployed at any time point (OR 6.6, 95% CI 1.37, 63.51). Delayed receipt was also significantly associated with having other children in the household at any time point, compared to no other children (1–2 other children OR 4.54, 95% CI 1.52–13.55, ≥3 other children OR 4.31, 95% CI 1.29, 14.37). Being born premature (<37 weeks gestation) was significantly decreased the odds of delayed vaccine receipt at 2 months of age (OR 0.15, 95% CI 0–0.96). There was likely to be insufficient power to statistically support other potential associations and multivariate regression was therefore not performed.

**Table 5:** Characteristics associated with delayed receipt of DTP at 2, 4 and 6 months of age

Characteristic	2 months			4 months			6 months			Any delayed		
	Delayed: n/N (%)	OR (95% CI)	*p*-value	Delayed: n/N (%)	OR (95% CI)	*p*-value	Delayed: n/N (%)	OR (95% CI)	*p*-value	Delayed: n/N (%)	OR (95% CI)	*p*-value
Sex												
Male	16/64 (25.0)			29/61 (47.5)			28/52 (53.8)			36/65 (55.4)		
Female	11/62 (17.7)	0.65 (0.27–1.53)	0.32	19/59 (32.2)	0.52 (0.25–1.10)	0.09	22/53 (41.5)	0.61 (0.28–1.32)	0.21	25/61 (41.0)	0.56 (0.28–1.13)	0.12
Care type												
Both parents	17/69 (24.6)			28/67 (41.8)			30/59 (50.8)			33/68 (48.5)		
Single parent	9/48 (18.8)	0.71 (0.25–1.89)	0.60	19/44 (43.2)	1.06 (0.46–2.44)	1.00	18/38 (47.4)	0.87 (0.35–2.13)	0.90	27/50 (54.0)	1.24 (0.56–2.76)	0.69
Other^a^	1/8 (12.5)	0.44 (0.01–3.85)	0.79	1/8 (12.5)	0.20 (0.00–1.72)	0.21	2/7 (28.6)	0.39 (0.03–2.63)	0.48	1/7 (14.3)	0.18 (0.00–1.61)	0.18
Primary Carer												
Mother	25/103 (24.3)			42/98 (42.9)			42/86 (48.8)			52/104 (50.0)		
Other^b^	2/16 (12.5)	0.45 (0.05–2.17)	0.48	5/15 (33.3)	0.67 (0.21–2.10)	0.49	5/12 (41.7)	0.75 (0.22–2.54)	0.64	5/15 (33.3)	0.50 (0.16–1.56)	0.23
Child care												
No	22/90 (24.4)			35/84 (41.7)			34/71 (47.9)			43/90 (47.8)		
Yes	5/35 (14.3)	0.52 (0.18–1.49)	0.22	13/35 (37.1)	0.83 (0.37–1.86)	0.65	15/33 (45.5)	0.91 (0.40–2.08)	0.82	17/35 (48.6)	1.03 (0.47–2.25)	0.94
Education level of mother												
Did not finish high school	10/51 (19.6)			19/49 (38.8)			23/45 (51.1)			27/51 (52.9)		
High school	13/56 (23.2)	1.24 (0.44–3.53)	0.83	21/53 (39.6)	1.04 (0.47–2.30)	0.93	18/45 (40.0)	0.64 (0.28–1.47)	0.29	24/56 (42.9)	0.67 (0.31–1.43)	0.30
Post-school qualification^c^	3/15 (20.0)	1.02 (0.16–4.92)	1.00	6/14 (42.9)	1.18 (0.36–3.95)	0.78	6/12 (50.0)	0.96 (0.27–3.42)	0.95	7/15 (46.7)	0.78 (0.25–2.47)	0.67
Education level of father												
Did not finish high school	14/57 (24.6)			21/54 (38.9)			25/48 (52.1)			30/56 (53.6)		
High school	8/31 (25.8)	1.07 (0.34–3.22)	1.00	14/30 (46.7)	1.37 (0.50–3.72)	0.64	12/26 (46.2)	0.79 (0.30–2.05)	0.63	15/31 (48.4)	0.81 (0.31–2.14)	0.81
Post-school qualification^c^	1/14 (7.14)	0.24 (0.01–1.89)	0.28	4/13 (30.8)	0.70 (0.14–2.93)	0.84	5/10 (50.0)	0.92 (0.24–3.59)	0.91	4/13 (30.8)	0.39 (0.08–1.60)	0.23
Employment status of mother												
Employed^d^	1/15 (6.7)			3/14 (21.4)			3/12 (25.0)			2/14 (14.3)		
Unemployed	26/109 (23.9)	4.35 (0.60–192.46)	0.23	45/104 (43.3)	2.77 (0.68–16.40)	0.20	46/92 (50.0)	2.97 (0.68–18.14)	0.18	58/110 (52.7)	6.60 (1.37–63.51)	0.01
Employment status of father												
Employed^d^	11/53 (20.8)			19/49 (38.8)			19/43 (44.2)			22/52 (42.3)	1.74 (0.79–3.80)	0.17
Unemployed	13/51 (25.5)	1.31 (0.52–3.26)	0.57	22/49 (44.9)	1.29 (0.58–2.88)	0.54	24/43 (55.8)	1.60 (0.68–3.74)	0.28	28/50 (56.0)	0.73 (0.42–1.27)	0.27
Number of other children in household												
0	3/26 (11.5)			5/23 (21.7)			5/19 (26.3)			5/24 (20.8)		
1–2	17/68 (25.0)	2.53 (0.64–14.81)	0.25	30/66 (45.5)	3.00 (1.00–9.04)	0.05	31/59 (52.5)	3.10 (0.99–9.71)	0.05	37/68 (54.4)	4.54 (1.52–13.55)	0.01
3+	7/30 (23.3)	2.30 (0.45–15.49)	0.43	13/29 (44.8)	2.93 (0.85–10.02)	0.09	12/25 (48.0)	2.58 (0.71–9.37)	0.15	17/32 (53.1)	4.31 (1.29–14.37	0.02
Income level												
< $26,000	8/42 (19.0)			19/40 (47.5)			20/35 (37.7)			28/44 (63.6)		
$26,000–≤ $52,000	15/49 (30.6)	1.86 (0.64–5.79)	0.31	20/47 (42.6)	0.82 (0.32–2.08)	0.81	18/40 (45.0)	0.62 (0.22–1.68)	0.41	22/49 (44.9)	0.47 (0.19–1.16)	0.11
$52,000–≤ $78,000	3/21 (14.3)	0.71 (0.11–3.46)	0.93	6/19 (31.6)	0.52 (0.13–1.82)	0.38	8/18 (44.4)	0.61 (0.16–2.18	0.56	7/19 (36.8)	0.34 (0.09–1.16)	0.09
> $78,000	1/9 (11.1)	0.54 (0.01–5.13)	0.99	2/9 (22.2)	0.32 (0.03–1.98)	0.31	2/7 (28.6)	0.31 (0.03–2.21)	0.33	2/9 (22.2)	0.17 (0.02–1.03)	0.06
Father’s age at birth of child												
< 20 years	2/7 (28.6)			3/7 (42.9)			4/7 (57.1)			5/7 (71.4)		
20–29 years	16/76 (21.1)	0.67 (0.10–7.66)	0.95	28/71 (39.4)	0.87 (0.14–6.39)	1.00	32/64 (50.0)	0.75 (0.10–4.84)	1.00	37/76 (48.7)	0.38 (0.03–2.52)	0.45
≥ 30 years	8/38 (21.1)	0.67 (0.09–8.32)	0.99	16/37 (43.2)	1.01 (0.15–7.93)	1.00	12/29 (41.4)	0.54 (0.07–3.84)	0.74	17/38 (44.7)	0.33 (0.03–2.34)	0.38
Mother’s age at birth of child												
< 20 years	6/28 (21.4)			11/27 (40.7)			14/26 (53.8)			14/28 (50.0)		
20–29 years	16/67 (23.9)	1.15 (0.36–4.08)	1.00	25/63 (39.7)	0.96 (0.35–2.69)	1.00	27/55 (49.1)	0.83 (0.29–2.33)	0.87	34/67 (50.7)	1.03 (0.43–2.49)	0.95
≥ 30 years	4/29 (13.8)	0.59 (0.11–2.88)	0.68	11/28 (39.3)	0.94 (0.28–3.17)	1.00	7/22 (31.8)	0.41 (0.10–1.51)	0.21	11/29 (37.9)	0.61 (0.21–1.75)	0.36
Gestational age												
≥ 37 weeks gestation	26/108 (24.1)			44/103 (42.7)			44/91 (48.4)			55/108 (50.9)		
< 37 weeks gestation	0/15 (0.0)	0.15 (0–0.96)	0.04	3/14 (21.4)	0.37 (0.06–1.51)	0.21	4/11 (36.4)	0.61 (0.12–2.61)	0.67	4/15 (26.7)	0.35 (0.08–1.29)	0.13
Birthweight												
≥ 2500 grams	25/103 (24.3)			42/98 (42.9)			43/87 (49.4)			53/103 (51.5)		
< 2500 grams	1/20 (5.0)	0.17 (0.00–1.15)	0.08	5/19 (26.3)	0.48 (0.12–1.55)	0.27	5/15 (33.3)	0.51 (0.13–1.82)	0.38	6/20 (30.0)	0.40 (0.14–1.13)	0.09

^a^Other = shared, other relative, other non-family member;^b^Other = Father, Mother and Father, Grandparent, Grandparent and Father, Other non-family; ^c^Post-school qualification = Certificate, Diploma, Bachelor Degree; ^d^Employed = Full Time, Part Time, Casual

## Discussion

We examined immunisation coverage and timeliness in a cohort of urban Indigenous children. According to participants’ ACIR records, less than half of the children aged ≥ 7, 13 or 19-months were up-to-date at enrolment into the cohort study. Delays in vaccine receipt increased as infants aged and parent/guardian reporting of vaccination status was unreliable. Given the higher incidence of vaccine preventable diseases in Indigenous children [3], and the importance of herd immunity, our data provide further support for the need to identify evidence-based strategies to improve timeliness [17] among this population.

Our results are consistent with a Northern Territory (NT) cohort study [9] that reported coverage of the primary series (also at 7-months of age) as 45.2%; coverage in this NT cohort was lower amongst children living in urban areas compared to remote areas. The authors suggested the reasons for the regional differences were likely related to differences in health service delivery and/or population movement between urban and remote areas. The proportion of timely immunisation of DTP in our cohort also falls within the ranges reported in an analysis of immunisation from 31 low and middle income countries [1] in which the timeliness of DTP was 67% (11.6–89.3%) for the first dose and 41% (10.8–82.1%) for the third dose. The proportions of our cohort with delayed receipt of DTP3 (39.9%) and PCV3 (40.2%) are comparable to national timeliness data of these vaccines (38.2% and 40.5% for DTP3 and PCV3 respectively) [5], however delayed receipt of MMR at 12 months was higher in our cohort compared to national data (53.7% vs 40.3%).

We also found that immunisation coverage and timeliness decreased as children got older; the proportion of delayed receipt increased by 19 percentage points between the first and third dose of DTP. This is consistent with findings from a study of an urban NSW Indigenous cohort [10], the NT data [9] and international data [1].

Non-receipt was highest for rotavirus vaccines, likely due to the upper age restrictions of administering rotavirus vaccines, and for the HepA vaccine at 18-months of age. We also identified that rotavirus vaccines were received after the recommended upper age limit among some children, potentially increasing the infant’s risk of intussusception [18]. Hull et al. [19] predicted potential impacts of these strict age-limits which included lower coverage of other vaccinations due to non-vaccination of late presenters, and non-adherence to age cut-offs. Our results support both of these predictions. Greater attention needs to be given to appropriate and timely administration of rotavirus vaccinations and providers may need to be reminded of the importance of adhering to the age limits of vaccine receipt.

Previous research suggests Indigenous-specific vaccines have lower levels of uptake in their target population than universally-recommended vaccines [6]. This was evident in our study population with a greater proportion of children receiving the universally recommended varicella vaccine at 18-months, compared to the Indigenous-specific HepA vaccine. Proportions of receipt in this cohort also differed between the three vaccines due at 12 months. Fragmented vaccine administration has been identified in previous studies amongst both Indigenous and non-Indigenous children. Ferson et al., found that among a cohort of non-Indigenous children, 9.2% of fully-immunised children had received vaccines due at the same time on separate occasions [20]. Similarly, O’Grady et al. [9] found differences in coverage of 7vPCV and DTP over the time period in which the 7vPCV was introduced in Australia. Administration of vaccines on separate occasions is reported to be a significant predictor of vaccination delay [21]. This could have contributed to the high rates of non-receipt and/or delayed receipt in our study. Despite national recommendations approving simultaneous administration of multiple vaccines, research suggests fragmented vaccine administration is often related to the advice and practices of immunisation providers [20, 21]. Further research needs to be conducted to determine the extent to which this phenomenon currently occurs in Australia and to understand provider’s intentions if and when making these recommendations.

We found that parental report of their child’s immunisation status over-estimated the proportion of children up-to-date. Thus, in this community, and potentially other similar communities, immunisation status should be confirmed by another source. Possible reasons as to why parents may believe their children are up-to-date could include a lack of parental understanding of what vaccines are required, a lack of personal vaccination records and/or communication issues between parents and health providers.

Despite the significant body of evidence indicating the need to improve timeliness among urban Australian Indigenous children, there is a lack of studies evaluating interventions to do this. One Australian study found that the distribution of personalised calendars to parents through health clinics was an effective and culturally appropriate way to improve timeliness of the primary series [22]. Identifying risk factors associated with non-timeliness may inform appropriate intervention points. We found that having other children in the household and a mother who was unemployed were potential predictors of delayed receipt of DTP at 2, 4 and/or 6-months, whereas prematurity was associated with a decreased odds of delayed vaccine receipt. . Living in a household with multiple children has previously been identified as a predictor of vaccination delay in Australia and internationally [1, 21, 23, 24], suggesting additional support needs to be provided for these families. While it could be presumed that being an unemployed mother may allow for greater time to have children vaccinated, unemployed mothers may be more likely to lack other resources such as finances, transport and social support. Other studies [21, 23] have identified single-parent households and low levels of parental education as predictors of delayed vaccine receipt, however we were unable to demonstrate these associations with the available data.

The main strength of this study is the use of ACIR records as the primary measure of a child’s immunisation status. ACIR records were retrievable for 97% of children we recruited. The allowance of 3 months lag between when the last vaccine in the cohort was due and the data of retrieval of ACIR records reduced the likelihood that incomplete immunisation records were due to reporting or processes delays. We did not employ the third-dose assumption that is routinely used to determine national immunisation coverage in this study. While this has been previously reported as a valid assumption [16, 25], these studies were conducted over a decade ago making their current applicability questionable, particularly given changes over time from a paper-based submission system to an electronic one. We could not validate the third dose assumption with personal health or clinic records as many of the study children received health care and vaccines at clinics other than our study site, particularly the one third (44/140) that were born prior to the opening of the clinic in July 2011. By using the 3rd dose assumption our analyses would have included an additional 21 vaccines administered between 8 kids. Hull and McIntyre [25] calculated that use of the third dose assumption increased the proportion of children fully immunised by 11–12%. While this would have raised the proportion of children fully immunised in our population to 59.6, 47.3 and 30.5% at 7, 13 and 19 months respectively, these levels remain noticeably below national levels.

Our analyses of potential predictors of timeliness and coverage were limited by the sample size and that child characteristics were collected at enrolment, not at the time vaccines were administered. It is possible that several of these factors would have changed between vaccine receipt and enrolment; however our results are consistent with other studies [1, 26]. Nevertheless comprehensive prospective studies are needed to confirm predictors of timely immunisation amenable to intervention. Finally, due to the retrospective nature of this study and the use of ACIR records, we were unable to assess to what extent early or delayed vaccine receipt was an intentional decision made by the immunisation provider and/or family.

## Conclusions

Immunisation coverage and timeliness in this population during the first 18-months of life was inadequate, placing children at increased risk of acquiring vaccine preventable infections during infancy and childhood. High quality trials, conducted in several settings to account for the diversity of Australian Indigenous communities are urgently needed to identify culturally appropriate, effective and sustainable strategies to improve immunisation targets in children.

This will also assist in highlighting subpopulations most in need of improvement and in monitoring change over time. Our data suggest that families with multiple children, low income or when the mother is unemployed should be specifically targeted for interventions such as intensive follow-up, home visiting and other support mechanisms. Further research needs to be conducted into the extent and cause of fragmented vaccine administration, as well as predictors of delayed receipt in a larger study.

## Abbreviations

7vPCV: 7-valent pneumococcal conjugate vaccine
ACIR: Australian Childhood Immunisation Register
ARI: Acute respiratory illness
DTP: Diphtheria-tetanus-pertussis
HepA: Hepatitis A
HepB: Hepatitis B
Hib: Haemophilus influenza type b
MenC: Meningococcal C
MMR: Measles-mumps-rubella
NT: Northern Territory
PCV: Pneumococcal conjugate vaccine
Rota: Rotavirus
